# The Complex Effects of Light on Metabolism in Humans

**DOI:** 10.3390/nu15061391

**Published:** 2023-03-14

**Authors:** Asuka Ishihara, Amber B. Courville, Kong Y. Chen

**Affiliations:** National Institute of Diabetes and Digestive and Kidney Diseases, Bethesda, MD 20892, USA

**Keywords:** light, metabolism, circadian rhythm, sleep, melatonin

## Abstract

Light is an essential part of many life forms. The natural light–dark cycle has been the dominant stimulus for circadian rhythms throughout human evolution. Artificial light has restructured human activity and provided opportunities to extend the day without reliance on natural day–night cycles. The increase in light exposure at unwanted times or a reduced dynamic range of light between the daytime and nighttime has introduced negative consequences for human health. Light exposure is closely linked to sleep–wake regulation, activity and eating patterns, body temperature, and energy metabolism. Disruptions to these areas due to light are linked to metabolic abnormalities such as an increased risk of obesity and diabetes. Research has revealed that various properties of light influence metabolism. This review will highlight the complex role of light in human physiology, with a specific emphasis on metabolic regulation from the perspective of four main properties of light (intensity, duration, timing of exposure, and wavelength). We also discuss the potential influence of the key circadian hormone melatonin on sleep and metabolic physiology. We explore the relationship between light and metabolism through circadian physiology in various populations to understand the optimal use of light to mitigate short and long-term health consequences.

## 1. Introduction

Light has a profound impact on most, if not all, species on Earth. In diurnal animals such as humans, natural daylight exposure usually induces wakefulness, and night leads to sleep; this has been the case throughout most of our evolutionary history. The adoption of artificial light in our modern society has led to several advantages, including extended human activity into dark hours, enabling control over the amount of light and extent of human activity occurring throughout the day. However, the deviation from naturally occurring light–dark cycles has introduced adverse effects on human health, including disturbance in sleep and circadian rhythm [1,2,3], along with increased risk for obesity [4,5] and metabolic disorders [1,6,7,8], which have been growing in prevalence worldwide. These interactions between sleep and circadian rhythm have been described previously [9], with additional factors built into this model, including the role of peripheral oscillators in regulating metabolism [9,10]. Numerous studies have sought to understand the mechanism of light exposure and metabolism in animal models [1,11]. However, the physiological impacts on humans are still limited. This review will focus on how light influences both sides of the energy balance equation, with an emphasis on humans, by (1) exploring the role of light in human metabolism via the distinct properties of light and (2) examining how exogenous melatonin intake from food sources functions as a potential key factor influencing sleep and metabolism.

## 2. Sleep and Circadian Impact on Metabolism

**Figure 1:**
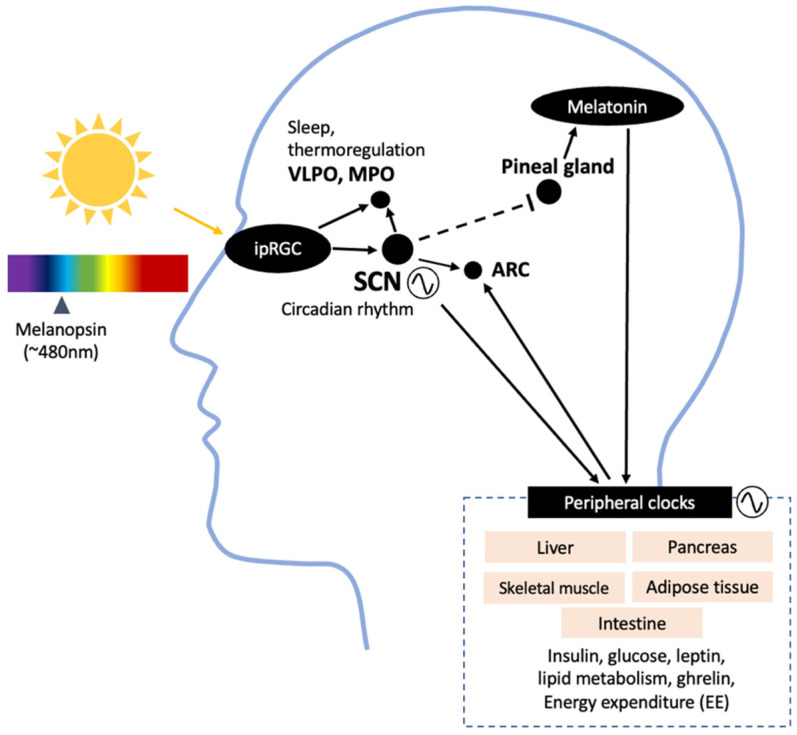
Projection sites of ipRGCs, showing an overview of the areas in the brain receiving input from light and involved in metabolic regulation, indicated by human and animal studies. SCN, suprachiasmatic nucleus; VLPO, ventrolateral preoptic nucleus; MPO, medial preoptic area; ARC, arcuate nucleus; EE, energy expenditure.

Light is detected by the photoreceptors in the retina, including the rods, cones, and intrinsically photosensitive retinal ganglion cells (ipRGCs). The basic function of a rod is to detect light levels, while cones identify color, spatial detail, and motion. In addition to these image-forming photoreceptors, melanopsin-expressing (sensitive to 480 nm light) ipRGCs are mainly responsible for the non-visual effects of light, such as circadian entrainment and sleep–wake regulation. Photic input from the ipRGCs travels through the retinohypothalamic tract (RHT) to transmit information to the suprachiasmatic nucleus (SCN), the central circadian pacemaker in the hypothalamus (Figure 1). Among many projection sites involved in regulating energy homeostasis, such as the arcuate nucleus (ARC), the SCN controls the output to the pineal gland, secreting melatonin [12]. Light information from the ipRGCs also projects to the ventrolateral preoptic area (VLPO) regulating sleep; the medial preoptic area responsible for temperature regulation; and the medial amygdala and lateral habenula, which regulate mood [2,13,14]. Although detailed functions of the ipRGCs are beyond the scope of this review, four subtypes of ipRGCs are known to exist in humans, each with distinct characteristics and projection destinations [15]. This emphasizes the complex role of ipRGCs in multiple areas of the brain affecting physiological and behavioral responses.

The endogenous circadian rhythm is determined by the daily behavior of the hormone melatonin, core body temperature, and rest–wake activity cycles [16]. Melatonin is the current gold standard for assessing the circadian rhythm as it directly reflects the rhythm in the SCN. Accordingly, melatonin secreted from the pineal gland is also light-sensitive and is suppressed during the day and secreted in the dark to help promote sleep by acting on the circadian rhythm. Human sleep is composed of non-rapid eye movement (NREM) and rapid eye movement (REM) sleep. NREM sleep is further divided into three stages, from shallowest to deepest: N1, N2, and N3, or slow-wave sleep (SWS). Sleep and energy metabolism are also linked, such that energy expenditure is lower during SWS and increases during wake after sleep onset, a brief period of arousal during sleep [17,18], suggesting energy conservation may be at play during sleep. Additionally, daily rhythms of hormone concentrations linked to metabolism are directly driven by the SCN, including insulin, glucagon, and corticosterone [19,20,21,22]. Aside from this, circadian rhythms are indirectly exhibited in almost all physiological functions, including energy metabolism (energy expenditure and substrate utilization) [23], via the SCN.

## 3. Properties of Light Affecting Metabolism

The central circadian clock in the SCN is modulated by the exogenous stimulus of light–dark exposures. Some of the main characteristics of light that concern sleep and the circadian rhythm are its intensity, duration, exposure timing, and wavelength. While increasing the dosage of light exposure in the evening through intensity and duration impacts sleep and circadian activity, other aspects of light, such as the timing of light exposure and spectral composition, have more specific targets. It is important to note that physiological attributes are linked, but not limited to, a combination of these properties of light. Other aspects include color temperature, flickering, spatial distribution, prior light history, and type of light (light-emitting diode vs. organic-light-emitting diode), all of which influence human physiology [24].

### 3.1. Intensity of Light

Natural outdoor light intensities vary greatly. Direct midday sunlight ranges from 20,000 to 100,000 lux [25]. Illuminance levels, often expressed in photopic lux, vary by outdoor location from 9300 lux in the open playground to 1500 lux under a big tree in the shade to 395 lux between buildings [26]. In comparison, the overall indoor artificial light intensity levels range from 14 to 430 lux depending on the proximity to the window [26]. The recommended minimum indoor light condition during the day is around 500 lux at eye level and 80 lux before sleep [27].

Spending time sleeping or awake under high-intensity light at night has indeed been shown to have a negative physiological impact on animals. In mice, continuous illumination during the dark phase increases body mass and reduces glucose processing without changing total food intake or activity [28]. In humans, under the constant routine protocol (method to study endogenous circadian rhythm by minimizing the effect of external factors), 5 h of light exposure at 0.03, 180, 1260, and 9500 lux showed a dose-dependent response to the phase shift of the core body temperature rhythm [29]. Similarly, suppression of melatonin and increased alertness from EEG activity was observed from increased intensity of light at night [30,31]. These findings in humans suggest the potential secondary influence of high light intensities at night on metabolism through the shift in circadian rhythm.

The relationship between light intensity and metabolic health has also been documented in epidemiological studies. In one study, bedroom brightness was significantly associated with an increased risk of high BMI and obesity in women (n = 113,000; 47.2 ± 13.6 yrs) [32]. In 513 older individuals with and without diabetes (diabetes: 32 females, 37 males, 72.4 ± 6.5 yrs; non-diabetes: 243 females, 201 males, 72.8 ± 6.5 yrs), ambient light intensity in the evening before bed from 17.5 to 37.6 lux was associated with a 51% increase in the prevalence of diabetes [33]. Artificial light at night from indoor and/or outdoor light during sleep was also significantly associated with an increased risk of weight gain and obesity in women (n = 43,722; 55.4 ± 8.9 yrs) [4]. In a cross-sectional study of 528 older Japanese individuals (281 females; 72.8 ± 6.5 yrs), those sleeping in rooms with >3-lux light tended to have a higher body weight, BMI, waist circumference, triglyceride level, and low-density lipoprotein cholesterol level compared to those sleeping in rooms at <3 lux [7]. Sleeping in a room with light (>3 lux) also resulted in a greater increase in waist-to-height ratio in 1110 older individuals (590 females; 71.9 ± 7.1 yrs) [34]. A prospective study conducted by Obayashi and colleagues also suggested that light at night (>5 lux) increases the incidence of diabetes in affected individuals compared to those sleeping with light at <5 lux (n = 678; 376 females; 70.6 ± 6.6 yrs) [35]. In a controlled study, light intensities well below the threshold in the dim light range (<10 lux) also altered nighttime sleep. An in-laboratory study among healthy men (n = 23; 22.3 ± 2.7 yrs) undergoing 5-lux and 10-lux light during sleep resulted in increased wake after sleep onset, increased N1, decreased N2, and increased REM sleep [36]. Similarly, a follow-up study with the same light conditions conducted in females (n = 25; 23.9 ± 3.0 yrs) showed an increase in wake after sleep onset and REM sleep, along with decreased total sleep time and sleep efficiency [37]. Although dim light melatonin onset did not differ between 5 and 10 lux of light at night, the acute reduction in total sleep time suggests the eventual consequence on metabolism.

Photic stimulation also impacts insulin regulation [38]. In a recent randomized controlled study in 20 healthy adults (room light condition: females = 8, males = 2, 26.6 ± 4.3 yrs; dim condition: females = 6, males = 4, 26.8 ± 5.2 yrs), sleeping in a room at 100 lux compared to <3-lux dim light for one night resulted in higher measures of insulin resistance and an elevated heart rate, suggesting a potential impact on glucose metabolism via the sympathetic nervous system pathway [39]. Similarly, mild sleep deprivation combined with nocturnal light exposure during sleep (600 lux) in healthy men (n = 8; 21.1 ± 0.9 yrs) increased postprandial insulin levels, suggesting insulin resistance, and disrupted plasma melatonin and cortisol levels. Moreover, light exposure at night increased glucagon-like peptide-1 (GLP-1), a gastrointestinal hormone facilitating insulin secretion. This increase in GLP-1 was not seen in sleep-deprived participants under the dark condition, indicating a direct impact of light on glucose homeostasis [40]. Moreover, bright light exposure also influences taste perception. Healthy individuals (9 females, 29 ± 6 yrs; 5 males, 32 ± 6 yrs) who were exposed to bright light (10,000 lux) for 30 min showed lower recognition of sucrose (sweet taste) compared to those under dim light exposure (20 lux) [41], which could lead to an increase in intake of sweet-tasting food after exposure to bright light.

In summary, until artificial light became a part of our lives, humans were exposed to natural outdoor bright light during the day (~9000 lux) and dimmer light at night. Deviating from this natural light intensity range, such as reducing daylight exposure and increasing illuminance during the dark cycle, alters sleep and metabolic regulation. It is important to note that light intensity is often measured in absolute metrics (lux, irradiance) and not weighted to the photopic sensitivity of the human eye. Melanopic lux integrates spectrum and intensity information and is closer to the illuminance perceived by the ipRGCs [42]. Additionally, interindividual variability in response to evening light needs to be considered further to understand the impact of light intensity on metabolism [43].

### 3.2. Duration of Light

Similar to intensity, the duration of light exposure also affects sleep and circadian rhythm in a dose-dependent manner. Under a constant routine protocol in 39 healthy young adults (16 females; 22.2 ± 3.6 yrs), a single session of high-intensity light exposure at 10,000 lux for 0.2 h, 1.0 h, 2.5 h, and 4.0 h suppressed melatonin and shifted the circadian rhythm in a dose-dependent manner [44]. In terms of the effect of light duration on metabolism in 48 children (25 females; 4.8 ± 0.4 yrs), increased duration of light exposure above the threshold of 200 lux was a predictor for increased BMI after controlling for sleep duration, sleep timing, and activity [45]. For instance, children (n = 2761; 1356 females; 1–4 yrs) who spent more time viewing television had a higher risk of being overweight [46]. A meta-analysis confirmed that extended screen time from electronic devices was positively associated with overweight/obesity in adolescents [47]. These observational studies suggest a link between the duration of light exposure and potential consequences on metabolism.

However, the nature of light exposure is far more complex. Circadian phase shifts have also been observed to occur at millisecond flashes of non-continuous, intermittent light exposure in the evening. In seven healthy individuals (one female; 18–48 yrs), 60 min of 2 msec pulses compared to the dark control environment led to a significant phase delay in the circadian rhythm [48]. Consistently, when various ranges of intermittent light pulses were tested (2.5 to 240 s pulses) in 39 healthy individuals (14 females; 26.4 ± 5.1 yrs), compared to continuous light with the same spectral content and intensity, intermittent light pulses were effective in phase delaying the circadian system, but in a nonlinear fashion [49]. These findings emphasize the complex nature of light exposure duration and how alterations in duration patterns may be reflected in metabolism. Nonetheless, under continuous light exposure, increasing the duration of exposure during nighttime disrupts sleep and phase delays the circadian system, altering metabolism in humans.

### 3.3. Exposure Timing

The timing of light exposure greatly influences the magnitude and direction of the circadian rhythm. Depending on the timing of light exposure, for instance, early or late evening, the circadian system may phase delay or advance [50].

#### 3.3.1. Morning Light Exposure

Bright light exposure shortly after awakening in the morning has been commonly used as an effective treatment among individuals with seasonal affective disorder (SAD) and winter depression to induce phase advance in the delayed circadian rhythm, potentially alleviating adverse metabolic states [51]. Gaist and colleagues investigated the current state of energy consumption among patients with SAD under normal light conditions (<500 lux) compared with bright light therapy (2500 lux) for 2.5 h in the morning for at least nine days. Resting metabolic rate (RMR), measured in 10 patients with SAD (7 females; 39.1 ± 5.8 yrs), had a higher baseline RMR compared to 9 healthy individuals (5 females; 38.9 ± 8.9 yrs). After the light treatment, the RMR in SAD patients was lowered compared to their baseline. This result may be attributable to changes in appetite and body weight before and after the light treatment in patients with SAD [52]. In a study conducted by Putilov and Danilenko, morning light treatment (2500 lux) for two hours for a week among 61 female patients resulted in a higher respiratory rate, oxygen consumption rate, and energy expenditure, possibly due to the activation of the sympathoadrenal system, potentially accompanied by an elevated heart rate [53]. A reduction in body weight and depressive response were also observed in patients with SAD after light treatment [53]. A more recent study by Ivanova and colleagues, however, revealed that 30 min of morning bright light treatment (4300 lux) for a day in 10 females (18–65 yrs) did not significantly alter oxygen consumption or carbon dioxide production compared to red light conditions (250 lux) [54]. The influence of winter depression also affects the eating habits of individuals with seasonal bulimia nervosa, which was suggested to improve with 30 min of bright light therapy [55,56]. Although studies conducted to examine the metabolic state of patients with depressive symptoms have been inconclusive, there is optimism that mood alleviation due to bright light therapy through serotonergic pathways could potentially improve metabolism. Continued investigation is necessary for therapeutic application among these individuals.

Epidemiologic studies have reported that individuals with type 2 diabetes have a high risk of developing depression [57]. Previous studies have indicated that morning bright light exposure may impact metabolism in a clinical population. Two case studies reported a reduction in insulin dose after phototherapy in insulin-dependent diabetic patients with winter depression [58,59], suggesting a positive impact on glycemic control. Additionally, morning light therapy at 10,000 lux for four weeks in 42 patients with depression and type 2 diabetes (21 females; 60.1 ± 9.8 yrs) showed no significant effect on insulin sensitivity [60]. A case study of four overweight women (46–54 yrs) treated with two-hour phototherapy (1500 lux) for ten days in the morning resulted in weight loss and improved mood in three of the women, suggesting the potential influence of light on the melatonin–serotonin system and carbohydrate metabolism [61].

Studies conducted on morning bright light exposure in individuals without depression have been less consistent. Among eight healthy, lean young men (21–24 yrs), five-hour morning bright light (4000 lux) exposure did not affect fasting or postprandial plasma glucose levels, while in eight older men with type 2 diabetes (54–63 yrs), fasting and postprandial glucose levels, as well as postprandial triglyceride level, increased after morning light exposure [62]. However, in a randomized clinical trial among 34 overweight women (20–54 yrs), a 45 min morning light treatment (1300 lux) for three weeks induced a reduction in percent body fat, body mass, and appetite [63]. The combined effect of exercise and bright light exposure in the morning has also been examined to explore the potential influence of mood and metabolic alleviation. A controlled laboratory study among 25 overweight individuals and those with obesity undergoing six weeks of morning bright light treatment (5000 lux) combined with moderate exercise showed a significant reduction in body fat compared to exercise without light treatment [64]. These results warrant further research on potential clinical implications in individuals with obesity.

Ambient bright light treatment has also been shown to be effective in improving sleep in young [65,66] and older adults [67], and individuals with dementia [68]. Collectively, morning bright light treatment may be a viable non-invasive method to treat individuals with delayed circadian rhythm and sleep disorders, as well as metabolic and mood disorders.

#### 3.3.2. Daytime Light Exposure

Light exposure during daytime in various animals has been referred to as the “dead zone” of the phase–response curve due to the lack of light-induced resetting of the circadian rhythm during daytime exposure [69]. However, it is unclear whether such a “dead zone” exists in humans, perhaps due to differences in activity periods for diurnal and nocturnal animals [50,70,71]. Since the term “dead zone” was coined, only a few studies have addressed the potential influence of daytime light exposure on metabolism in humans.

In a randomized controlled study among 15 healthy individuals (7 females; 23.3 ± 3.4 yrs), 14 h of daytime light exposure did not lead to significant changes in 24 h energy expenditure or respiratory quotient, an index of fat and carbohydrate oxidation [72]. Dim light during the day (10 lux) and bright light in the evening (1250 lux), compared to daytime bright and evening dim light, blunted the usual increase in postprandial glucose in 14 insulin-resistant older adults (4 females; 67 ± 6 yrs) [73]. Additionally, in 27 females with SAD (34.8 ± 10.3 yrs), daytime light treatment (2500 lux) between 14.00 and 16.00 h for a week was observed to reduce body weight and increase oxygen consumption compared to pre-treatment [74]. Furthermore, daytime light exposure has been implicated to alter the digestive, endocrine, and autonomic nervous systems. Dim light exposure (80 lux) during the day in 11 healthy young females (19–25 yrs) resulted in higher postprandial breath hydrogen excretion (indicative of carbohydrate malabsorption) and lower postprandial electrogastrography (indicative of gastric myoelectric activity) compared to under bright light conditions (5000 lux) [75].

The amount of light exposure during the day can also impact metabolism during sleep, emphasizing the importance of daytime light conditions. A study by Harmsen and colleagues showed that exposure to dim light during the day and bright light in the evening decreased sleeping metabolic rate compared to baseline [73]. These studies on daytime light exposure, along with epidemiological findings [76], have crucial implications for sleep and overall energy metabolism.

#### 3.3.3. Evening Light Exposure

Extended exposure to light during the dark phase has been shown to alter metabolism. In animals, constant light reduces the amplitude of the circadian rhythm in the SCN, increases food intake, decreases energy expenditure, and increases body weight, along with reducing insulin sensitivity [77,78]. The extended exposure to light induces arrhythmicity in the circadian rhythm and the peripheral clocks. Similarly, in humans, evening and extended light exposure are both associated with increased body weight, higher BMI [8], and risk of obesity [79]. Additionally, objective measurement of sleep and light levels using actigraphy showed a positive correlation between mean light exposure timing and BMI, as well as with the midpoint of sleep [80].

Studies have been conducted extensively on the shift-working population to understand the link between light and metabolism. These studies have consistently shown an association between working in shifts or during the night and metabolic impairments [81]. Shift working is associated with an increased risk of overweight and obesity [82,83,84], diabetes [85], metabolic syndrome [86,87], and abdominal obesity, all of which are indicative of lipid disturbance [88]. These individuals often face multiple unfavorable conditions, including circadian disruption, sleep deprivation, and exposure to light periods at an atypical time, leading to irregular food intake.

Evening light exposure also influences whole-body energy metabolism, especially during sleep. In 10 healthy young men (mean ± SE: 25.7 ± 0.65 yrs), exposure to polychromatic white light (1000 lux) for 4 h prior to sleep significantly increased respiratory quotient and reduced fat oxidation during sleep compared to dim light (<10 lux) conditions [89]. Analogous to this finding, bright light exposure (10,000 lux) 3 h before sleep among ten healthy men (mean ± SE: 26 ± 1 yrs) also significantly increased their respiratory quotient and decreased fat oxidation during sleep, along with leading to suppressed melatonin compared to the control light conditions (<50 lux) [90]. Bright light exposure (10,000 lux) for 3 h before sleep in eight healthy men (mean ± SE: 26 ± 1 yrs) also altered urinary characteristics [91]. Specifically, urinary carnosine, anserine, 4-amino-3-hydroxybutyric acid (GABOB), and ornithine decreased after bright light exposure, suggesting an attenuated ability to recover from fatigue during sleep [91]. Along with the suppression of salivary melatonin after exposure to evening bright light [92], urinary melatonin metabolites were correlated less with respiratory quotient after exposure to LED light [89], suggesting the role of melatonin in metabolic regulation [93]. Additionally, evening exposure to the bright light of 2000 lux compared to 20 lux in nine healthy females (18–20 yrs) significantly lowered breath hydrogen excretion, suggesting a worse effect on carbohydrate digestion [94]. Thus far, the majority of the studies linking evening light exposure and energy metabolism have been conducted among healthy young men. Future studies on diverse populations are needed.

#### 3.3.4. Evening Light Exposure and Food Intake

The endogenous circadian rhythm is entrained primarily by the external light–dark cycles, in which light information is obtained and transmitted directly to the SCN. Although the central oscillator is phase-locked to light–dark cycles, the timing of food intake can also influence individual circadian rhythms in the peripheral organs [95]. Thus, the timing of food consumption may impact the SCN secondarily through peripheral clocks, further complicating the alignment of the circadian rhythm throughout the body. The relationship between eating at night and the increased risk of obesity has been suggested by several observational studies in shift- and night-working populations. Additionally, shifting the timing of food intake has consequential effects on metabolism. The shift in dinner timing from 19:00 to 22:30 h in healthy men increased postprandial plasma glucose levels [96]. To understand this, it is crucial to consider the role of light in circadian rhythm and feeding behavior.

In 16 healthy individuals (8 females (22.2 ± 2.6 yrs) and 8 males (22.8 ± 3.5 yrs)), exposure to bright light (>500 lux) in the evening and while asleep significantly increased plasma glucose and insulin levels compared to dim light exposure (<5 lux), implicating glucose intolerance and insulin insensitivity [97]. For night shift workers and individuals with night eating syndrome, a large portion of total energy intake occurs after dinner, which may contribute to health consequences [98]. Preference for certain types of food and dietary intake among shift workers has been examined in simulated and free-living conditions. After a simulated night shift in 16 young individuals (8 females; 20.1 ± 1.4 yrs), a significant preference for high-fat food (protein: 14.9%; fat: 64.8%; carbohydrate: 20.3%), as opposed to low-fat food (protein: 36.4%; fat 6.4%; carbohydrate: 57.2%), was apparent in the morning following the shift, despite being presented an equal number of calories for either choice [99]. In a free-living study, 24 female shift workers (25–47 yrs) underwent an ad libitum 14-item test meal buffet to investigate food preference. Although the total calories consumed during the test meal were not significantly different between the groups, day workers consumed more protein than night workers (16.0 ± 5.7% vs. 11.8 ± 4.1%) [100]. Subjective hunger and desire to eat (by visual analog scale) were higher after bright light exposure (>500 lux) in 10 healthy individuals (5 females; 22.4 ± 3.2 yrs) compared to dim light exposure following an isocaloric evening meal [101]. Therefore, nocturnal bright light exposure, combined with disrupted sleep and circadian misalignment, may negatively impact metabolic regulation.

The timing of light exposure has been studied for three main time points: morning, daytime, and evening. Although most studies focus on reporting light information during the intervention period, there is a lack of information on light conditions outside of this exposure period. Nevertheless, it is possible that the timing of light exposure may alter and extend feeding behavior at night and impact metabolism, as observed in rodent models [1]. Additionally, the general view of restricting food intake before sleep has been challenged due to new perspectives on food intake at night. One study has reported that small amounts of protein-rich beverages demonstrated a positive impact on muscle protein synthesis at nighttime in healthy men [98]; thus, food intake at night may contribute positively to metabolism depending on the amount and composition of the food consumed. Understanding the effect of the food composition and the amount consumed may provide ways to minimize metabolic consequences when food consumption is inevitable at nighttime. Taken together, light exposure at appropriate times plays a crucial role in maintaining the circadian rhythm, whereas deviating from this may lead to irregular rhythm and altered metabolism and feeding behavior.

### 3.4. Spectral Composition of Light

The spectral composition of light also plays a crucial role in regulating metabolism. Spectral composition impacts ipRGCs’ function as melanopsin, photopigment expressed in ipRGCs, is most sensitive to short wavelengths of light at 480 nm. Exposure to blue light, but not green (~550 nm) light, in the evening, or the use of LED devices that emit high levels of blue light, reportedly influences sleep, circadian rhythm, and thermoregulation [102,103,104,105].

Morning light exposures of up to 60 lux of red (633 nm), green (532 nm), and blue (475 nm) light after 5 hrs of sleep restriction in 11 individuals (6 females; 27.4 ± 8.7 yrs) significantly increased leptin concentrations. However, ghrelin concentration decreased under red and green light exposures, suggesting differences in hunger variability due to sleep restriction depending on light conditions [106]. Contrarily, in 19 healthy adults (11 females; 28 yrs), blue-enriched light (468 nm) did not alter leptin and ghrelin compared to dim light exposure [86]. Blue-enriched light, regardless of the timing of exposure, reduced subjective sleepiness and increased insulin total area under the curve, HOMA-IR, and HOMA-IR area under the curve compared to dim light [86]. Evening exposure to blue-enriched light compared to dim light led to a higher peak glucose, suggesting its influence on insulin secretion [86]. Additionally, in nine healthy young men, alterations in sleeping energy metabolism under two hours of evening exposure to monochromatic blue light (465 nm) resulted in decreased energy expenditure, carbon dioxide production, and oxygen consumption after waking up [107].

The wavelength of light has been shown to influence individuals’ motivation to eat food, stimulating their appetite and willingness to consume the food presented to them [108,109]. The speed of eating, amount consumed, and preference for healthy or unhealthy food have also been associated with an ambient light environment [110,111]. A randomized trial of 112 participants (50 females and 62 males; 18–58 yrs) exposed to either white, yellow, or blue light (ranging from 9.0 to 13.5 lux) during breakfast demonstrated that blue light decreased the amount of food consumed in men but not in women, while other colors did not alter food intake [112].

Electronic devices such as smartphones, tablets (including e-readers), and laptops are widely used around the world. The use of these devices at night has been questioned due to their short wavelength of blue-light-emitting displays, which may impact sleep, circadian rhythm, and metabolism. Some studies have addressed the impacts of electronic devices that contribute to suppressed melatonin and increased arousal [102] and the use of “Night Shift” features to filter out blue light at night [113]. To investigate whether this feature affects metabolism, healthy young individuals (seven females and six males; 29 ± 5 yrs) underwent three trials consisting of using an iPad with and without the Night Shift mode on and a paper book under dimmed room light for one hour before bedtime. Despite no difference in the illuminance level of the room between the conditions, the use of an iPad without the Night Shift feature resulted in a non-significant but moderate suppression of leptin levels at night, while more comparable results were observed after the use of Night Shift and hard-copy books [114]. Together, these studies emphasize the importance of considering the spectral content of light on metabolic physiology, with special attention to the short wavelength, as it is most sensitive to the melanopsin-expressing ipRGCs.

### 3.5. Other Properties of Light-Influencing Metabolism

A recent epidemiology study reported that midday solar ultraviolet (UV) exposure (between 1100 h and 1300 h) increased food-seeking behavior and food intake in men but not in women. A similar finding of weight gain was observed solely in male mice [115]. These properties of light affecting peripheral regions of the body in a sex-dependent manner emphasize the importance of understanding the influence of light on individual organs.

Artificial lighting is also characterized as color temperature, describing the visual “warmness” and “coolness” based on the spectral power distribution of light. Generally, increased color temperature is composed of more power in the blue spectrum. Light with a 1900 K color temperature containing a spectrum ranging from 500 to 650 nm of yellow–orange–red light (1900 K) in 40 individuals (18–25 yrs) induced significantly higher levels of melatonin compared to light exposure to color temperatures at 3000, 4000, and 6600 K [116]. However, differences were not observed between color temperatures at 3000 and 5700 K in 16 individuals (9 females; 23.8 ± 3.1 yrs) [117]. Glutamate secretion, which is indicative of cognitive ability, was also promoted by 1900 K after two hours of light exposure [116]. The influence of color temperature on thermal and metabolic changes has been studied in relation to ambient temperature. Effects of color temperatures at 3000, 5000, and 7500 K at 500 lux in 11 males (19–26 yrs) revealed that exposure to the ambient temperature of 15 °C reduced the skin and rectal temperature more so at 3000 K than at 5000 K or 7500 K, but with no significant difference in metabolic heat production [118]. Contrarily, in 16 females (22.2 ± 2.4 yrs) exposed to 2700 K and 5800 K at 55 lux under ambient temperature at 26 °C, greater self-assessed shivering was indicated for high color temperature (5800 K) compared to low color temperature (2700 K) [119]. Thermal physiological parameters of skin temperature, core body temperature, and energy expenditure did not alter with the self-assessed shivering after exposure to different color temperatures [119]. Greater thermal comfort was achieved at 2700 K compared to 6500 K (n = 32; 23 females; 23.9 ± 3 yrs), which is in line with the hue-heat hypothesis that the blue-rich spectrum is associated with a cooler sensation [120]. A simulated study on color temperature (2800 K, 4000 K, 5000 K, and 6500 K) under a range of ambient temperatures (21, 24, 27 °C) showed minimal changes in skin and core body temperature under a neutral color temperature at 5000 K, while any deviation was predicted to increase physiological responses, contributing to an altered perception of ambient temperature [121]. The color temperature of light is intricately linked to perceived temperature (thermal comfort) and thermal physiological responses that may also impact metabolism.

Finally, the duration, rather than the intensity of light exposure, has a more pronounced impact on the magnitude of light-induced phase delays [122]. Although specific properties of light may have a stronger impact on physiology, the magnitude of the outcome may be augmented when these components are combined. Additionally, in most free-living situations, multiple light properties, including those mentioned above as well as those not necessarily focused on this review, are at play simultaneously. Thus, we emphasize the importance of considering a wide range of light properties that potentially influence metabolic regulation in humans.

## 4. Melatonin’s Contribution to Metabolism

In humans, SCN activity is most often measured based on endogenous melatonin levels. In young adults, melatonin production at nighttime is typically around 10–80 μg [123]. One study also reported that the peak melatonin concentration ranged from 2 to 84 pg/mL in 170 individuals (85 females; 26.2 ± 6.0 yrs) [124]. Although melatonin depends primarily on photic input for its secretion, its receptors are expressed throughout the body in extra-pineal sites [125], including, but not limited to, the retina, gastrointestinal tract, bone marrow, skin, and lymphocytes [126]. Through these extra-pineal sites, the physiological influence of melatonin extends beyond sleep and circadian regulations, affecting thermoregulation and energy metabolism, as previously studied in animals [127,128].

The relationship between melatonin and metabolism has been well documented through pinealectomy studies in animals where a lack of melatonin induces diabetogenic syndrome and metabolic abnormalities, such as diminished glucose tolerance, reduced hepatic and muscular glycogenesis, and insulin resistance [127,128,129,130]. Observational studies in humans have also shown a reduction in melatonin amplitude with blunted rhythms in individuals with type 2 diabetes [131,132]. Nighttime melatonin also interacts with insulin in individuals with metabolic syndrome [133]. Thus, melatonin plays a crucial role in regulating energy metabolism and energy balance.

### 4.1. Exogenous Melatonin Administration

Earlier studies have reported the effectiveness of exogenous melatonin in improving sleep and circadian rhythm disorder in individuals undergoing jet lag or shift work, and those with visual impairments [125]. The administration of exogenous melatonin has been explored to understand its function in human metabolism, which was shown to impact lipid and glucose metabolism [134,135]. In women with obesity, a negative association was observed between the intake of exogenous melatonin supplements and BMI [136]. A 3-week randomized crossover trial of melatonin treatment in 36 individuals with type 2 diabetes and insomnia (25 females; 46–77 yrs) improved sleep efficiency and wake after sleep onset without affecting glucose and lipid metabolism [137]. In addition, the influence of melatonin on lipoproteins in 15 normolipidemic postmenopausal women (48–55 yrs) after receiving an oral dose of 6 mg melatonin at night for two weeks showed a significant increase in plasma triglyceride and VLDL cholesterol levels [135].

In the shift-working population, melatonin administration can alleviate circadian misalignment [138] and improve sleep, alertness [139,140], and energy intake [138]. In terms of its effectiveness in preventing weight gain, in a randomized crossover trial, the administration of melatonin (3 mg) in shift-working individuals on non-shift-working days (27 females; 37.1 ± 5.9 yrs) resulted in a reduction in body weight, BMI, waist circumference, and hip circumference without changes in caloric intake, which was accompanied by 20% reduction in circadian misalignment [138]. In a randomized crossover trial in 27 female shift workers with a BMI over 25 kg/m^2^ (37.1 ± 0.6 yrs), 12 weeks of melatonin administration (3 mg) compared to a placebo during free-living conditions resulted in comparable total energy intakes, macronutrient distributions, types of food consumed, and meal timings, suggesting independent functions of melatonin and food intake [141].

The potential role of light, combined with the influence of exogenous melatonin, has been studied in healthy individuals. Melatonin administration (2 mg) in nine healthy males (26 ± 1.3 yrs) under light exposure (>500 lux) at night increased leptin levels compared to the light condition without melatonin administration, which was accompanied by lower subjective hunger and appetite [142]. Significant reductions in postprandial plasma glucose, insulin, and triacylglycerol levels were observed in the melatonin-administered condition, indicating enhanced glucose tolerance and insulin sensitivity [143]. Contrarily, the acute administration of melatonin (5 mg) for two consecutive days in 21 healthy females (24 ± 6 yrs) resulted in an impairment of glucose tolerance, suggesting decreased insulin sensitivity in the evening [144]. This conflicts with the studies conducted by Albreiki and animal studies showing that melatonin ameliorates glucose and insulin resistance [145,146]. Although mixed results have been reported in response to melatonin administration in humans, light conditions were different in each study, and thus future studies incorporating or delineating the added influence of light are necessary.

### 4.2. Melatonin-Enriched Food on Sleep

Melatonin expresses a photoperiodic mechanism exhibiting a daily rhythm in vertebrates. This characteristic is observed outside the animal kingdom, such as in unicellular alga, *Gonyaulax polyedra* [147], and other food plants and medicinal herbs [148,149,150]. Additionally, melatonin is also present in plant and plant products such as fruit, vegetables, and wheat [149], as well as in beverages including coffee, tea, beer, and wine [151,152]. Specifically, cranberries, coffee, and some herbs contain high levels of melatonin [153,154,155]. In addition, melatonin is detected in lamb, beef, pork, chicken, and fish [156]. However, to what degree this impacts human physiology is still unknown and needs to be considered. For instance, the reported melatonin concentration level varies from pg/g to mg/g depending on the food sources [152]. Although melatonin is detected in a wide variety of food sources, the effect of melatonin-containing food sources on objective sleep quality in humans has been reported in only a few randomized control studies [123,157]. Whether foods rich in melatonin affect human physiology (sleep and metabolism) and to what extent need to be examined carefully.

#### 4.2.1. Fruits

Sour cherry or Montmorency cherry has been reported to have beneficial effects on sleep, which may be attributed to its high concentration of melatonin and tryptophan, the essential amino acid that is the precursor of endogenous melatonin and serotonin [158]. In 20 healthy young adults (10 females; 26.6 ± 4.6 yrs), consumption of tart cherry juice for seven consecutive days significantly elevated total urinary 6-sulfatoxymelatonin and increased time in bed, total sleep time, and sleep efficiency, measured using actigraphy [159]. Clinical implications of the role of cherry-based products have been examined mainly in older individuals and those with insomnia. In a crossover trial consisting of 15 individuals (7 females; 71.6 ± 5.4 yrs) with insomnia, tart cherry juice (two 8-ounce servings) reduced wake after sleep onset [160]. Additionally, sleep duration and subjective sleep quality improved in eight individuals (five females; 68 ± 9.2 yrs) consuming cherry juice before bedtime for 14 days [161]. Cherry-based products consumed twice a day for three days led to increased sleep efficiency in 30 individuals (15 females; 20–85 yrs) [162] as well as in 12 males (35–85 yrs) [163]. Studies on pineapple, oranges, and bananas indicated elevated serum melatonin concentrations after the consumption of these fruits [164].

#### 4.2.2. Milk

Milk is also known to contain tryptophan and melatonin naturally. However, the amount may vary greatly (10–40 ng/L), and its impact on circulatory melatonin levels and sleep quality is difficult to quantify [165]. In one study examining the effect of melatonin-enriched milk (1 ng melatonin/sachet, 58.4 ng tryptophan/sachet) and regular milk (0.1 ng melatonin/sachet, 46.9 ng tryptophan/sachet) on adults (73 females; 21–69 yrs old) for two weeks showed a significant difference in the sleep satisfaction scale and daytime sleepiness between the groups, especially in individuals between 20 and 30 years old [166]. In children, evening consumption of milk-based drinks did not improve total sleep time or sleep efficiency measured using actigraphy. It did, however, lessen nocturnal awakening and better memory recall [167].

The potential benefits of probiotics such as lactobacilli and bifidobacteria for stress and mental health have been explored [168]. Daily intake of milk fermented with Lactobacillus casei strain Shirota (LcS) in comparison to non-fermented placebo milk among medical students during a period of heightened stress from exams showed that subjective sleep improved after LcS treatment [168]. Moreover, single-channel EEG recorded shorter sleep latency and increased delta power in the LcS-treated group compared to the placebo [168]. Mice fed milk at night had shortened sleep onset and prolonged sleep duration, but with no significant changes in EEG waves [169].

#### 4.2.3. Grain and Other Food Sources

Studies have suggested high melatonin content in rice and corn [152]. Whole grains, barley, and rice also improve sleep quality in humans. Associations have been drawn from rice [170] and whole-grain consumption [171] and higher subjective sleep quality. Consumption of cereal enriched with tryptophan (60 mg tryptophan in 30 g cereal) two times per day in 35 individuals (26 females; 55–75 yrs) increased sleep efficiency, sleep time, and decreased nocturnal activity, as measured by accelerometry [172].

The hypnotic effect of this molecule has been previously studied [173]. A study examining the influence of food-derived tryptophan on sleep showed that tryptophan-rich de-oiled gourd seed combined with glucose improved subjective and objective measures of insomnia compared to carbohydrate foods [174]. A study examining the combined effects of tryptophan supplementation during breakfast and daytime light exposure (5000 lux) in 33 healthy men (22 ± 3.1 yrs) showed it promoted melatonin secretion in the evening compared to a tryptophan-lacking breakfast under dim light conditions (<50 lux) [175]. Similar findings were reported in an epidemiological study on college students [176], along with a study on the dietary intake of tryptophan on metabolic syndrome [177].

Aside from these food sources, a considerable amount of melatonin has been detected in meat, fish, egg, cereal, fruits, vegetables, legumes, and nuts [152]. As such, other food sources rich in melatonin levels would need to be investigated to understand their effects on sleep and metabolism, combined with light exposure. Finally, food sources provide an aggregate of various nutrients. How certain nutrients in food interact with each other (i.e., melatonin and caffeine in coffee) needs to be further explored. Well-controlled studies are necessary to assess the relationship between melatonin in food and sleep, circadian rhythm, and metabolism.

## 5. Variability in Response to Light

Endogenous melatonin is known to vary depending on age and sex. Melatonin concentration at night declines with age and differences in daytime and nocturnal levels become less pronounced [178]. The sex difference is also observed in melatonin. Among 18 females (26.7 ± 0.8 yrs) and 16 males (24.5 ± 0.7 yrs), the amplitude of melatonin rhythm was significantly greater [179,180] with an earlier phase in females [179]. Additionally, men were shown to have greater light sensitivity, especially to blue-enriched light in the evening, than women [181]. The underlying mechanism of sex-related differences in light sensitivity influencing sleep, circadian rhythm, and metabolism remains to be fully established.

Sleep and circadian disorders are common among blind individuals, with severity depending on the degree of light perception [182,183]. Many visually impaired individuals have a free-running circadian rhythm with periods lasting longer than 24 h [184], while those with complete loss of ocular perception of light have a greater concentration of daily plasma melatonin compared to sighted individuals, due to reduced nighttime suppression of melatonin [185]. Individuals with limited light perception have an abnormal circadian rhythm, which has been treated with daily administration of melatonin [182,183]. Since visually impaired individuals tend to have higher levels of BMI [186], assessing the potential benefits/challenges of melatonin administration on metabolism, along with a possible link with cancer [187] in blind individuals, will be of great importance going forward.

Considering how exogenous melatonin may impact individuals based on their sensitivity to melatonin may help us to understand the effect size of melatonin intervention through food sources. Orally administered melatonin comes in much higher quantities, i.e., between 1 and 5 mg, than that found in food products [188]. Studies focusing on how exogenous melatonin may coincide with the function of endogenous melatonin would be an essential point to focus on, especially among older individuals with reduced endogenously secreted melatonin. Future studies integrating light exposure and its impact on sleep and metabolism may extend our understanding of diet interventions containing melatonin-rich food.

## 6. Conclusions

Light has several properties influencing human sleep, circadian rhythm, and metabolic physiology. Natural light gradually changes throughout the day, while many questions regarding photic entrainment and metabolic implications influenced by the various components of light remain.

The increased use of artificial light sources in our daily lives reduces the dynamic range of natural light exposure from the day–night cycles, thus altering the natural circadian stimulus, which impacts energy intake and energy expenditure. The body of evidence from epidemiological studies suggests a general link between artificial light exposures and the risk of obesity and diabetes, which has been supported by mechanistic studies in animal models. However, association studies are multifactorial, and mice are nocturnal. Well-controlled clinical studies in humans are needed to quantify the effect size of the different components of light on human physiology and delineate acute vs. chronic effects. Expanding the population to explore the role of light in physiological variabilities such as age, sex, adiposity, and clinical population would give insight as to how light may influence individuals differently.

Adjusting the properties of light or altering the circadian hormone melatonin via the use of exogenous supplementations or consuming specific foods rich in melatonin and tryptophan may help minimize physiological consequences undergone by shift workers and individuals exposed to light at unwanted times. It could also be used as a treatment to readjust the misaligned circadian rhythm in clinical populations to help tackle metabolic disorders. With artificial light becoming widely popular, considering an optimal way to implement light into our daily lives would be crucial in minimizing negative health consequences induced by irregular light exposure deviating from the natural light–dark cycles.

## Data Availability

Not applicable.
